# An occupational therapy lens on microbiota–gut–brain modulation in ASD and ADHD

**DOI:** 10.3389/fresc.2026.1807954

**Published:** 2026-03-23

**Authors:** Anas, Syed Suhaib Ahmed, Inamul Hasan Madar

**Affiliations:** 1 Department of Occupational Therapy, Yenepoya Faculty of Allied and Healthcare Professions, Yenepoya (Deemed to be University), Mangalore, Karnataka, India; 2 Department of Pharmaceutics, Yenepoya Pharmacy College & Research Centre, Yenepoya (Deemed to be University), Mangalore, Karnataka, India; 3 Centre for Integrative Omics Data Science (CIODS), Yenepoya (Deemed to be University), Mangalore, Karnataka, India

**Keywords:** autism spectrum disorder, gut brain axis, gut brain microbiota axis, occupational participation, occupational therapy

## Abstract

Research on the microbiota gut brain axis has expanded rapidly, with growing interest in its potential relevance to neurodevelopmental conditions such as autism spectrum disorder and attention-deficit hyperactivity disorder. Systematic reviews increasingly report associations between gut microbial alterations and behavioral features in these populations, yet causal mechanisms remain unconfirmed and translational relevance for rehabilitation remains limited. Much of the existing literature prioritizes microbial composition, inflammatory markers, and neurochemical correlates, with comparatively little attention to everyday functioning, participation, and lived experience. This Perspective argues that microbiota gut brain interactions should be conceptualized within rehabilitation sciences as contextual physiological modulators, referring to internal bodily processes that shape readiness and engagement without determining occupational performance outcomes, and influencing occupational readiness and regulatory capacity rather than acting as etiological drivers of participation restrictions. Drawing on ecological and occupation-centered models of function, including the International Classification of Functioning framework and occupational therapy theories of performance and participation, we propose a reframing that preserves functional orientation while integrating emerging neurophysiological insights. The paper outlines implications for rehabilitation assessment, intervention planning, and interdisciplinary collaboration, and advances programmatic directions for future research that prioritize participation-based outcomes and ecological validity, defined here as the extent to which outcomes reflect real-world participation and everyday functioning rather than laboratory-based or symptom-only change. Repositioning microbiota gut brain research within a participation-focused rehabilitation framework may enhance translational value and support function-oriented, interdisciplinary care for children with autism spectrum disorder and attention-deficit hyperactivity disorder.

## 1 Introduction

This Perspective advances an occupational therapy framing of MGBA as a contextual physiological modulator of occupational readiness and participation, rather than an etiological driver of ASD or ADHD. We argue that rehabilitation sciences should integrate MGBA insights through participation-based outcomes and ecological models to avoid biological reductionism. Research on the microbiota–gut–brain axis (MGBA) has heightened in recent years, explicating a complex, bidirectional communication network among the gut microbiome, immune system and central nervous system. Systematic reviews increasingly suggest that alterations in gut microbial diversity are associated with autism spectrum disorder (ASD) and attention-deficit/hyperactivity disorder (ADHD), although causal mechanisms remain speculative and unconfirmed (1, 2). Despite this growing body of evidence, most MGBA research remains embedded within a biomedical archetype that privileges microbial composition, inflammatory markers and neurochemical correlates over considerations of everyday functioning. Consequently, the lived implications of these physiological processes for participation, routines and sensory regulation are underexplored. For occupational therapists (OTs), this gap presents both a challenge and an opening: to translate emerging physiological insights into an understanding of how internal regulatory systems may influence occupational readiness, engagement and performance. This Perspective reframes gut–brain interactions as contextual physiological modulators that influence, but do not determine, participation and daily functioning within an occupational therapy epistemology. Such a framing maintains the profession's focus on occupational performance within organic systems of environment, health and behavior while resisting the reduction of human occupation to its biological substrates.

Biomedical frameworks often position gut dysbiosis as a causal factor in the behavioral and cognitive features observed in autism spectrum disorder (ASD) and attention-deficit/hyperactivity disorder (ADHD). However, the current evidence base remains methodologically limited, characterized by small sample sizes, methodological variability, and heterogeneous findings across studies (3, 4). From an occupational therapy lens, gut-brain interaction is more suitably conceptualized through the disciplinary paradigms of performance capacity, occupational readiness (the individual's physiological, emotional, and regulatory capacity to engage in occupations at a given moment) and participation (involvement in life situations across home, school, and community contexts), which are foundational to theoretical models such as the Person-Environment-Occupation-Performance (PEOP) framework and the Model of Human Occupation (MOHO). Within this standard, biological processes are not regarded as primary reasons of dysfunction but as contextual aspects that influence an individual's energy regulation, arousal and interoceptive awareness. These physiological dimensions form the conditions under which occupational performance unfolds, yet they do not determine it. Looking the microbiota–gut–brain axis in this manner allows occupational therapists to integrate emerging neurophysiological knowledge into holistic reasoning while also maintaining focus on function, participation and the broader ecological context of human occupation.

Although systematic reviews report associations between gut microbial profiles and ASD/ADHD-related features, the current MGBA evidence base remains methodologically limited. Key constraints include small and heterogeneous samples, cross-sectional designs, inconsistent microbiome sequencing pipelines, dietary and medication confounding, and substantial between-study variability in outcomes. Reported associations are often modest and non-replicated, limiting causal inference and clinical translation. Emerging mechanistic pathways involving immune signaling, neuroendocrine modulation, and vagal afferent activity provide biologically plausible accounts of gut–brain communication; however, these pathways remain incompletely specified and insufficient to support deterministic or etiological interpretations in rehabilitation contexts. Accordingly, we conceptualize MGBA influences as hypothesis-generating physiological modulators of occupational readiness rather than causal drivers of participation restriction. This article is a conceptual Perspective rather than a systematic or scoping review. Its scope is limited to pediatric rehabilitation contexts in ASD and ADHD and focuses specifically on implications for occupational therapy reasoning, assessment, and interdisciplinary collaboration. The paper selectively integrates MGBA literature relevant to functional modulation and participation while not attempting comprehensive coverage of microbiome methodologies, dietary interventions, or pharmacological modulation.

## 2 The gut–brain axis through an occupational therapy lens

### 2.1 Gut–brain signaling and occupational function

Children with autism spectrum disorder (ASD) and attention-deficit/hyperactivity disorder (ADHD) frequently display fluctuating stimulation levels and sensory modulation challenges that interrupt participation in everyday activities. Systematic reviews have identified associations between gastrointestinal discomfort, behavioral dysregulation and heightened emotional reactivity in these populations, suggesting that internal physiological states may contribute to observable patterns of engagement and self-regulation (1, 5). Gut–brain signalling, operating through immune, endocrine and vagal pathways, is hypothesized to influence stress tolerance and autonomic balance; conceptually, this may shape a child's capacity to engage meaningfully in daily routines and occupations, without implying causal determination.

### 2.2 Clinical and functional implications for occupational therapy

For occupational therapists, these insights have practical implications for both assessment and intervention. When children show irritability, distractibility or sensory defensiveness, clinicians, specifically Occupational Therapists, can consider whether physiological discomfort, fatigue or hunger may be the contributing factors. Intervention should remain grounded in an occupation-centered approach that emphasizes interoceptive awareness, energy pacing, and optimal sensory–environmental fit rather than attempts to directly modify biological mechanisms. Such an approach maintains the focus on adaptive participation and functional engagement which align with the foundational occupational therapy principle that variations in performance are best understood as the outcome of compound interactions among physiological, environmental and psychosocial systems, likewise as manifestations of a singular biological cause. Occupational therapy literature provides well-established frameworks for addressing interoception, sensory processing, routine stability, and participation in children with ASD and ADHD. Interoceptive awareness has been increasingly emphasized in pediatric OT as a foundation for self-regulation and engagement in daily occupations (6). Sensory processing and modulation differences in ASD and ADHD are widely documented within occupational therapy research, with intervention approaches emphasizing adaptive sensory–environmental fit rather than normalization of sensory responses (7). Routine-based interventions and family-centered coaching approaches have demonstrated relevance for promoting participation and occupational engagement across home and school contexts in neurodevelopmental conditions (8). Embedding MGBA-informed clinical reasoning within these established occupational therapy paradigms strengthens rehabilitation grounding and avoids positioning physiological modulation as a primary driver of participation outcomes.

For example, a child with ASD presenting with fluctuating irritability and withdrawal during school-based therapy sessions may demonstrate improved engagement when intervention timing is adjusted to periods of greater physiological comfort and routine stability. This adjustment does not presume biological causality but operationalizes physiological modulation as one contextual factor influencing occupational readiness.

### 2.3 Routine stability and participation

Stable daily routines encircling mealtimes, play, sleep and school participation provide the structural foundation for occupational development and psychosocial well-being. Emerging evidence is suggestive that disruptions within the gut-brain axis may subtly destabilize these routines which influence the coherence and predictability of daily occupations. Meta-analytic findings point towards the evidence that children with autism spectrum disorder (ASD) frequently experience functional gastrointestinal disorders that coincide with sleep disturbance and selective eating patterns (9, 10). Physiological disturbances like these may diminish routine stability and interfere with co-occupations within the family system that can ultimately be constraining participation across social and educational contexts. Analogous patterns have been reported in attention-deficit/hyperactivity disorder (ADHD), where variations in microbiome composition have been associated with emotional reactivity, inconsistent arousal regulation and sleep quality disruption (2, 5). Conceptually, these physiological rhythms may modulate a child's readiness for learning, sustained attention, and engagement in goal-directed activity, rather than directly determining participation outcomes.

### 2.4 Ethical scope and interdisciplinary collaboration

From an occupational therapy perspective, these insights highlight the significance of addressing the functional consequences of physiological modulation rather than seeking to alter biology directly. Therapists can promote participation by developing stability in daily routines, supporting caregiver–child co-regulation and adapting environmental conditions which enhance comfort and also predictability. Conceptualizing gut-brain interactions as contextual influences on occupational performance allows practitioners to focus on the practical aspects of participation while maintaining a commitment to function-oriented and evidence-informed care. Occupational therapists do not participate in the prescription or management of microbiota-targeted treatments which includes probiotic supplementation or specialized dietary interventions. However, an understanding of gut brain modulation can enrich clinical reasoning by elucidating how physiological comfort, digestive functioning and energy fluctuations may influence participation and engagement in daily occupations. Recognizing these physiological dimensions allows therapists to refine their approach to assessment, planning and collaboration without extending beyond their professional scope. Within this framework, occupational therapists can integrate several practice strategies. They can incorporate interoceptive mapping within sensory assessments to identify internal signals that affect arousal and self-regulation. Interventions may be scheduled in alignment with natural variations in energy, digestion or medication cycles to optimize readiness for participation. Collaboration with dietitians, physicians and other health professionals supports a shared understanding of how physiological factors intersect with occupational performance. In addition, therapists can provide caregiver education on co-regulation practices that mitigate distress, such as establishing predictable and sensory-sensitive mealtime or bedtime routines.

## 3 Discussion

This professional stance safeguards ethical boundaries while enhancing the depth of functional and interdisciplinary insight. The occupational therapist's primary focus remains on facilitating meaningful participation, promoting occupational justice and supporting adaptation within the contexts of everyday life. Despite the rapid expansion of research on the microbiota–gut–brain axis (MGBA), systematic reviews continue to identify a notable absence of participation-based and functional outcome measures within this body of work (3, 9). Contemporary MGBA research predominantly prioritizes microbial diversity indices, inflammatory markers, and symptom severity scales as primary outcomes, whereas rehabilitation sciences prioritize participation, role performance, and contextual functioning as indicators of meaningful change. This Perspective advances a novel reframing by explicitly positioning MGBA variables as contextual modulators of occupational readiness within established occupational therapy models (ICF, MOHO, PEOP), thereby extending prior physiological discussions into participation-oriented clinical reasoning. Unlike existing MGBA narratives that emphasize biological remediation, this contribution situates gut–brain insights within occupation-centered epistemology, strengthening translational relevance for rehabilitation practice.

### 3.1 Three propositions for rehabilitation-oriented MGBA research

- Proposition 1: MGBA studies in ASD and ADHD should include participation outcomes (COPM, SFA) as co-primary endpoints. For example, MGBA-informed trials evaluating dietary or microbiota-targeted adjuncts in pediatric neurodevelopmental services could incorporate COPM and SFA alongside microbial and symptom outcomes to capture functionally meaningful change.
- Proposition 2: Rehabilitation trials should model MGBA variables as moderators of occupational readiness, not causal determinants of participation. Conceptually, MGBA variables may moderate responsiveness to participation-focused interventions (e.g., sensory integration or routine-based coaching) rather than directly producing occupational change.
- Proposition 3: Interdisciplinary pediatric protocols should align intervention timing with physiological comfort and arousal rhythms. For instance, scheduling therapy sessions to align with postprandial comfort or stable arousal windows may improve engagement without assuming biological causality.

The majority of studies focus predominantly on microbial diversity, inflammatory biomarkers or symptom reduction with a very limited attention to how these physiological variables relate to the lived experience or even daily functioning. This narrow focus restricts the translational relevance of MGBA research for occupational therapy. Occupational therapists are uniquely positioned to address this gap by integrating validated participation-focused assessments into interdisciplinary study designs. Instruments such as the Canadian Occupational Performance Measure (COPM) and the School Function Assessment (SFA) can identify meaningful and relevant changes in engagement, performance satisfaction and contextual participation that are not reflected in the biological data alone. Incorporating these measures may extend the scope of MGBA research beyond symptom-focused outcomes and align it well with the International Classification of Functioning, Disability and Health (ICF) framework which prioritizes participation and environmental interaction as key indicators of well-being. Such an approach may improve the ecological validity, defined here as the extent to which outcomes reflect real-world participation and everyday functioning rather than laboratory-based or symptom-only change, of MGBA investigations by systematically assessing the limit to which modulation of physiological regulatory processes corresponds with quantifiable improvements in occupational task performance, functional outcomes and participation in everyday life aspects.

The gut–brain axis includes a multidimensional regulatory network that offers a valuable framework for elucidating the neurophysiological mechanisms underlying self-regulatory capacity, arousal modulation and adaptive engagement. However, uncritical integration of this construct into clinical reasoning risks biological reductionism, wherein complex occupational behaviors and participation patterns are wrongfully framed as linear or deterministic consequences of physiological signaling. Occupational therapy provides a critical epistemological counterweight by contextualizing emerging evidence on gut–brain interactions within models of occupation that prioritize participation, performance and environmental embeddedness. Reconceptualizing gut–brain dynamics as modulatory influences on occupational readiness instead of primary etiological drivers of dysfunction preserves the profession's principled commitments to function, agency and contextualized meaning-making. This systems-oriented perspective distinguishes the contributory role of physiological regulation while clearly resisting its favoring over social, environmental and experiential determinants of occupation. By integrating mechanistic understandings into physiological modulation with ecologically grounded assessment, intervention design and translational research, occupational therapists can advance integrative care models that link biological processes with lived experience. Such models support participation-centered, function-driven practice and embrace particular significance for enhancing meaningful engagement across home, school and community contexts for children with autism spectrum disorder and attention-deficit/hyperactivity disorder. All clinical implications proposed in this Perspective remain within the professional scope of occupational therapy and do not endorse or prescribe microbiota-targeted interventions; rather, they emphasize participation-focused reasoning and interdisciplinary collaboration grounded in existing rehabilitation practice standards

## Data Availability

The original contributions presented in the study are included in the article/Supplementary Material, further inquiries can be directed to the corresponding author.
